# The influence of waiting times and sociopolitical variables on public trust in healthcare: A cross-sectional study of the NHS in England

**DOI:** 10.1016/j.puhip.2024.100484

**Published:** 2024-03-06

**Authors:** H. Dorussen, M.E. Hansen, S.D. Pickering, J. Reifler, T.J. Scotto, Y. Sunahara, D. Yen

**Affiliations:** a University of Essex, United Kingdom; b Brunel University London, United Kingdom; c University of Amsterdam, the Netherlands; d University of Exeter, United Kingdom; e University of Glasgow, United Kingdom; f Kobe University, Japan

**Keywords:** Trust, Healthcare, Waiting times, Ethnicity, National Health Service (NHS)

## Abstract

**Objectives:**

This study aims to assess factors influencing public trust in the National Health Service (NHS) in England, focusing on the impact of waiting times in Accident & Emergency (A&E) departments and for GP-to-specialist cancer referrals.

**Study design:**

A cross-sectional survey-based research design was employed, covering the period from July 2022 to July 2023.

**Methods:**

Data were collected through YouGov surveys, yielding 7415 responses. Our analysis is based on 6952 of these responses which we were able to aggregate to 42 NHS Integrated Care Boards (ICBs) for A&E waiting times and 106 ICB sub-units for cancer referral times. Multiple regression analysis was conducted, with the dependent variable being trust in the NHS.

**Results:**

Waiting times for A&E and cancer referrals did not significantly affect trust in the NHS. However, other sociopolitical factors displayed significant influence. Specifically, being a member of an ethnic minority group, or having voted Conservative in the 2019 general election were associated with lower trust scores. Other variables such as age and local unemployment rate were also significant predictors.

**Conclusions:**

Our findings suggest that waiting times for healthcare services have no effect on public trust in the NHS. Instead, trust appears to be largely shaped by sociopolitical factors. Policymakers should therefore look beyond operational efficiency when seeking to bolster trust in the healthcare system.

## 1 Introduction

The Covid-19 pandemic created challenges to health care systems across the world. During this period citizens across the United Kingdom were encouraged to show their support for the NHS by clapping on a weekly basis [1]. A 99-year-old retired Army officer, Capt Thomas Moore (later Sir Thomas Moore) began fundraising for the NHS through walking his garden and took the country by storm [2]. These events were seen as a signal of strong support for the NHS and its services by the British people [2]. However, since 2022 the NHS has faced increased challenges with rising waiting times for patients – both in being seen at emergency departments (“A&E”), but critically also in whether treatments for diagnosed conditions and diseases are delivered in a timely fashion [3]. Waiting times are not a new challenge to the NHS [4], but they are now at a level where they are described as a crisis [3]. Delays in cancer treatment (mostly due to the COVID-19 pandemic) decreased survival rates [5,6]. Similarly, longer wait times at emergency departments are associated with increased patient mortality and generally negative outcomes for the patient [7]. Using the concept of trust as a lens, we explore links between waiting times and people's evaluation of public institutions.

Trust is a central concept within the social sciences [8,9] and concerns the confidence that citizens have in the ability of government and political institutions to execute their duties effectively [10]. Generalized trust refers to a presumption of good intentions in the absence of specific information about an institution, and serves as a social glue that binds citizens to their political system, encouraging participation and cooperation. It is influenced by cultural norms, social experiences, or societal trends [11]. Generalized trust can be essential in forming a stable political environment where citizens are willing to accept decisions made by authorities, even when these decisions may not align with their personal interests. In contrast to *generalized* trust*, specific* political trust is grounded in personal experiences, judgements, and knowledge about a particular political entity (e.g., a politician, a political party, or a specific government institution) [10,12]. Specific trust is based on perceptions of competence, integrity, fairness, and the degree to which it aligns with an individual's values or expectations. Specific trust can be dynamic, changing with evolving perceptions of performance, credibility, and responsiveness [13]. Repeated positive interactions can foster specific trust, while perceived failures or scandals can erode it [8].

The relationship between trust and welfare services is complex [14], but the importance of trust for the delivery of healthcare cannot be overstated [[15], [16], [17], [18]]. In fact, there has long been a call for much more research on the relationship between trust and healthcare [15]. Comparatively across Europe there are generally high levels of trust in the healthcare systems, however, trust is generally lower within populations who are older, female and unemployed [19]. There is also research demonstrating a positive relationship between perceived healthcare performance and trust in relevant institutions [20]. In fact, the link between performance of welfare state institutions and trust in them is well-established [21,22]. Given that nearly everyone either accesses healthcare themselves or will have close contact with someone who does it is reasonable to expect that they rely on such experiences when making general judgements about the services [10,23] In fact, there is a strong relationship between experience of healthcare and people's levels of trust [22,24]. Compared to other welfare services healthcare is different and generally has strong public support [25,26].

Turning from Europe generally to the specific case of the NHS and the UK, public trust in health care has been challenged over the years by a number of scandals and poor performance, specifically long waiting times [16]. How do the recent service delivery challenges that the NHS has faced affect support for this bedrock modern British institution? This research will help expand the general knowledge on the relationship between performance and trust, and can help inform the strategies taken by policy-makers to engage with the well-known challenges facing the NHS in England today and health services in general.

## 2 Methods

### 2.1 Analytical approach

We combine survey data with contextual data for key performance variables (namely local-level NHS waiting times) to perform regression models that allow us to examine relationships between these waiting times, and our key outcome measures, namely trust.

### 2.2 Survey details

Our survey data were collected by YouGov in a series of 13 monthly waves from July 2022 to July 2023. The survey was restricted to England. Respondents ranged in age from 18 to 98. In total, 7415 responses were received. Of these, we were able to successfully identify the NHS Integrated Care Board (ICB) and ICB sub-units of 6952 respondents, based on their postcode. These formed the basis of our sample. The 13 waves are pooled together for analysis, although in each wave the respondents form a nationally representative sample. We have not sampled for recent experience in using the A&E service or receiving or being close to someone receiving cancer treatment. These variables are covered by the contextual level variables discussed below.

### 2.3 Dependent variable

**Fig. 1:**
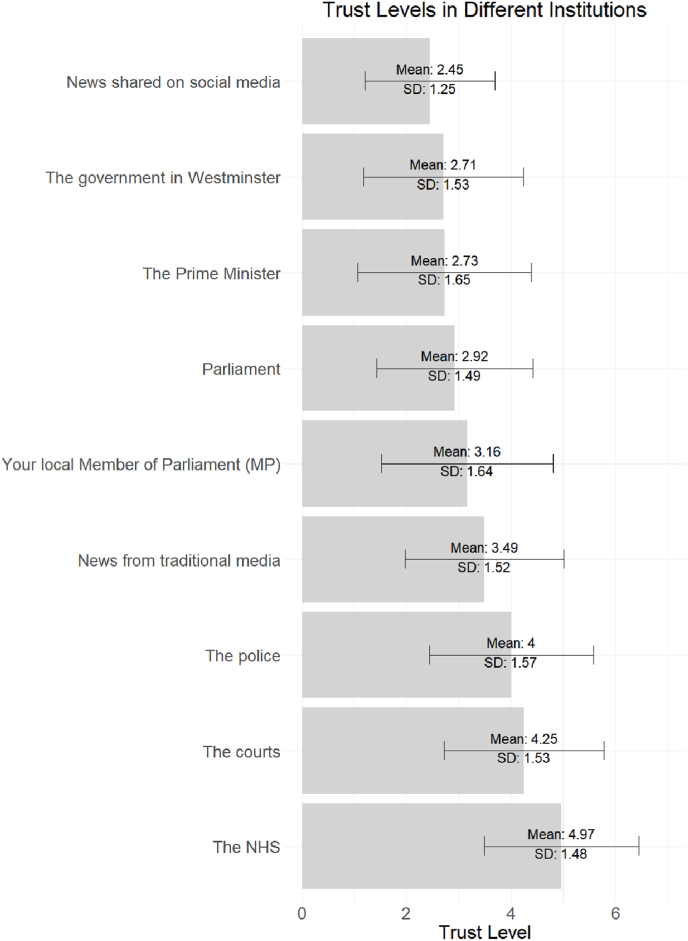
Levels of trust in nine different institutions.

Our main dependent variable is trust in the NHS. Across the 13 waves of our survey, respondents were presented with a matrix, to ask how much they trusted nine institutions (the Government in Westminster; the Prime Minister; their local Member of Parliament (MP); the NHS; the police; the courts; news from traditional media; and news shared on social media). Respondents were presented with a seven-point scale to measure their degree of trust, where 1 means “Not at all” and 7 means “Completely”. As can be seen in Fig. 1, the NHS is consistently more trusted by respondents than any of the other factors we have looked at in our surveys.

### 2.4 Independent variables

#### 2.4.1 Contextual-level measures

The most important contextual level variables in our analyses are measures that assess delays in the provision of NHS services: delays in Accident and Emergency (A&E) waiting rooms and delays in cancer referrals.

To calculate A&E delays we looked at the 42 Integrated Care Boards in the NHS in England. These 42 ICBs were the unit of analysis for the A&E delay measure. All types of A&E department were included (major, single speciality, minor injury units and other). To match the period of our survey, data were captured for July 2022 to July 2023, specifically looking at the proportion of people who had ‘waits of over 4 h for admission following decision to admit’ [27].

Our cancer wait time data is even more granular. The ICBs were further divided into ICB sub-units, of which there are a total of 106. Cancer referral delays were gathered at the level of these sub-units. Again, data were captured for the period July 2022–June 2023. The delays were measured by looking at the proportion of all referrals in the sub-unit which breached the “two week wait” between GP referral and appointment with a hospital specialist standard (a legal right since 2010).

In both instances, a script was written to map our survey respondents to the appropriate ICB and ICB sub-unit by using the first half of the respondents’ postcodes (the “outcode”).

#### 2.4.2 Individual-level survey measures

The survey data include responses about the socio-demographic characteristics of our respondents (in some cases we asked these questions in our survey, in other cases they come from profile variables collected and provided by YouGov). These sociodemographic variables include age (in real number of years), sex (male, female), place of residence (nine regions of England), level of education (completed higher education or not), ethnicity (member of ethnic minority or white) and income group (14 categories, from “under £5000” to “£100,000 and over” per annum).

#### 2.4.3 Regional-level measures

Also included are: a measure of the unemployment rate in the parliamentary constituency of each respondent; an individual-level measure of whether the respondent voted for the Conservative party in the 2019 British General Election; and the strength of support for the Conservative party in their constituency at the 2019 election.

Statistical methods.

A multivariate regression analysis using ordinary least squares (OLS) was conducted with all variables included and the levels of trust as the dependent variable. In addition to the main OLS analysis, we conducted a robustness check to assess the stability of our results. Specifically, we implemented an ordered logistic regression model using cumulative link models (CLM) to accommodate the ordinal nature of our dependent variable, trust in the NHS. This model also considers survey weights to correct for potential sampling biases. The results of this robustness check are reported in the Appendix.

## 3 Results

The descriptive statistics for the respondent-level variables are presented in Table 1, with region-level variables presented in Table 2. The distribution of survey respondents by Integrated Care Board and ICB sub-unit are presented in Table 3, Table 4.

**Table 1:** Descriptive Statistics of Survey Respondents

Factor	Category	%	Factor	Category	%
**Trust in the NHS**	1 (Not at all)	3.36	**Income**	Below £5000	8.88
	2	4.07		£5000 - £9999	10.16
Mean:	3	7.82		£10,000 - £14,999	14.15
4.97	4	16.53		£15,000 - £19,999	10.38
SD:	5	27.31		£20,000 - £24,999	12.51
1.48	6	27.16		£25,000 - £29,999	10.69
	7 (Completely)	13.74		£30,000 - £34,999	8.68
**Region**	North East	5.13		£35,000 - £39,999	5.69
	North West	13.28		£40,000 - £44,999	4.51
	Yorkshire and the Humber	10.47		£45,000 - £49,999	3.08
	East Midlands	9.65		£50,000 - £59,999	4.27
	West Midlands	10.01		£60,000 - £69,999	2.21
	East of England	12		£70,000 - £99,999	2.83
	London	12.62		£100,000 +	1.96
	South East	16.99	**Higher education**	Yes	41.56
	South West	9.86		No	58.44
**Gender**	Female	54.59	**Voted Conservative 2019**	Yes	36.46
	Male	45.41		No	63.54
**Ethnic minority**	Yes	14.04	**Age**	Mean: 50.91	Min: 18
	No	85.96		SD: 17.08	Max: 98

**Table 2:** Descriptive statistics of regions.

Factor	Mean	SD	Min	Max
**Cancer 2 week wait time breached in ICB sub-unit (%)**	20.85	12.14	0.8	63.12
**A&E more than 4 h wait in ICB (%)**	28.28	6.36	0	51.19
**Unemployment rate in constituency (%)**	3.52	1.65	1.04	11.29
**Conservative share in constituency (%)**	47.6	15.65	7.82	76.72

**Table 3:** Distribution of survey respondents by ICB.

ICB	#	ICB	#
Bedfordshire, Luton and Milton Keynes	113	Leicester, Leicestershire and Rutland	107
Birmingham and Solihull	125	Lincolnshire	141
Black Country	127	Mid and South Essex	153
Bristol, North Somerset and South Gloucestershire	105	Norfolk and Waveney	156
Buckinghamshire, Oxfordshire and Berkshire West	234	North Central London	165
Cambridgeshire and Peterborough	131	North East and North Cumbria	392
Cheshire and Merseyside	308	North East London	175
Cornwall and The Isles Of Scilly	86	North West London	160
Coventry and Warwickshire	125	Northamptonshire	101
Derby and Derbyshire	140	Nottingham and Nottinghamshire	167
Devon	164	Shropshire, Telford and Wrekin	69
Dorset	116	Somerset	90
Frimley	81	South East London	184
Gloucestershire	91	South West London	174
Greater Manchester	290	South Yorkshire	185
Hampshire and Isle Of Wight	236	Staffordshire and Stoke-on-Trent	104
Herefordshire and Worcestershire	105	Suffolk and North East Essex	149
Hertfordshire and West Essex	153	Surrey Heartlands	121
Humber and North Yorkshire	213	Sussex	234
Kent and Medway	208	West Yorkshire	294
Lancashire and South Cumbria	254

**Table 4:** Distribution of survey respondents by ICB-sub-unit.

Integrated Care Board	#	Integrated Care Board	#	Integrated Care Board	#	Integrated Care Board	#
Bedfordshire, Luton and Milton Keynes ICB M1J4Y	113	Greater Manchester ICB 01D	21	Lancashire and South Cumbria ICB 02G	9	Shropshire, Telford and Wrekin ICB M2L0M	69
Birmingham and Solihull ICB 15E	125	Greater Manchester ICB 01G	24	Lancashire and South Cumbria ICB 02 M	46	Somerset ICB 11X	90
Black Country ICB D2P2L	127	Greater Manchester ICB 01W	47	Leicester, Leicestershire and Rutland ICB 03W	18	South East London ICB 72Q	184
Bristol, North Somerset and South Gloucestershire ICB 15C	105	Greater Manchester ICB 01Y	33	Leicester, Leicestershire and Rutland ICB 04C	33	South West London ICB 36L	174
Buckinghamshire, Oxfordshire and Berkshire West ICB 10Q	106	Greater Manchester ICB 02A	18	Leicester, Leicestershire and Rutland ICB 04V	56	South Yorkshire ICB 02P	32
Buckinghamshire, Oxfordshire and Berkshire West ICB 14Y	58	Greater Manchester ICB 02H	32	Lincolnshire ICB 71E	141	South Yorkshire ICB 02X	34
Buckinghamshire, Oxfordshire and Berkshire West ICB 15A	70	Greater Manchester ICB 14L	48	Mid and South Essex ICB 06Q	51	South Yorkshire ICB 03L	34
Cambridgeshire and Peterborough ICB 06H	131	Hampshire and Isle Of Wight ICB 10R	28	Mid and South Essex ICB 07G	9	South Yorkshire ICB 03 N	85
Cheshire and Merseyside ICB 01F	11	Hampshire and Isle Of Wight ICB D9Y0V	208	Mid and South Essex ICB 99E	37	Staffordshire and Stoke-on-Trent ICB 04Y	12
Cheshire and Merseyside ICB 01J	6	Herefordshire and Worcestershire ICB 18C	105	Mid and South Essex ICB 99F	37	Staffordshire and Stoke-on-Trent ICB 05D	9
Cheshire and Merseyside ICB 01T	18	Hertfordshire and West Essex ICB 06K	66	Mid and South Essex ICB 99G	19	Staffordshire and Stoke-on-Trent ICB 05G	20
Cheshire and Merseyside ICB 01V	23	Hertfordshire and West Essex ICB 06 N	63	Norfolk and Waveney ICB 26A	156	Staffordshire and Stoke-on-Trent ICB 05Q	29
Cheshire and Merseyside ICB 01X	9	Hertfordshire and West Essex ICB 07H	24	North Central London ICB 93C	165	Staffordshire and Stoke-on-Trent ICB 05V	16
Cheshire and Merseyside ICB 02E	30	Humber and North Yorkshire ICB 02Y	48	North East and North Cumbria ICB 00L	43	Staffordshire and Stoke-on-Trent ICB 05W	18
Cheshire and Merseyside ICB 12F	53	Humber and North Yorkshire ICB 03F	30	North East and North Cumbria ICB 00 N	20	Suffolk and North East Essex ICB 06L	71
Cheshire and Merseyside ICB 27D	100	Humber and North Yorkshire ICB 03H	25	North East and North Cumbria ICB 00P	27	Suffolk and North East Essex ICB 06T	49
Cheshire and Merseyside ICB 99A	58	Humber and North Yorkshire ICB 03K	22	North East and North Cumbria ICB 01H	46	Suffolk and North East Essex ICB 07K	29
Cornwall and The Isles Of Scilly ICB 11 N	86	Humber and North Yorkshire ICB 03Q	47	North East and North Cumbria ICB 13T	61	Surrey Heartlands ICB 92A	121
Coventry and Warwickshire ICB B2M3M	125	Humber and North Yorkshire ICB 42D	41	North East and North Cumbria ICB 16C	88	Sussex ICB 09D	31
Derby and Derbyshire ICB 15 M	140	Kent and Medway ICB 91Q	208	North East and North Cumbria ICB 84H	81	Sussex ICB 70F	118
Devon ICB 15 N	164	Lancashire and South Cumbria ICB 00Q	23	North East and North Cumbria ICB 99C	26	Sussex ICB 97R	85
Dorset ICB 11J	116	Lancashire and South Cumbria ICB 00R	27	North East London ICB A3A8R	175	West Yorkshire ICB 02T	33
Frimley ICB D4U1Y	81	Lancashire and South Cumbria ICB 00X	32	North West London ICB W2U3Z	160	West Yorkshire ICB 03R	42
Gloucestershire ICB 11 M	91	Lancashire and South Cumbria ICB 01A	41	Northamptonshire ICB 78H	101	West Yorkshire ICB 15F	109
Greater Manchester ICB 00T	20	Lancashire and South Cumbria ICB 01E	20	Nottingham and Nottinghamshire ICB 02Q	13	West Yorkshire ICB 36J	74
Greater Manchester ICB 00V	26	Lancashire and South Cumbria ICB 01K	56	Nottingham and Nottinghamshire ICB 52R	154	West Yorkshire ICB X2C4Y	36
Greater Manchester ICB 00Y	21						

### 3.1 Regression analysis

The results of the ordinary least squares regression are shown in Table 5 Two models are presented: Model 1 includes respondents' personal income, while Model 2 excludes this variable, as almost a quarter of our respondents chose not to declare their income. Across both models, levels of trust in the NHS are positively influenced by age: using Model 1 as the baseline, for each year the respondent increases in age there is an average increase in trust of 0.008, or for each ten years a respondent ages, their trust increases by 0.08. Members of ethnic minorities have notably lower levels of trust in the NHS than white respondents. Those who stated they voted for the Conservative party at the 2019 British General Election have comparably lower levels of trust in the NHS than non-Conservative voters. There are no regional effects: levels of trust in the NHS are the same, no matter where people live in England, while having completed higher education, the regional share of votes for the Conservative party or the size of the respondent's NHS sub-unit had no significant effect. Most notably, neither the amount of time waiting for cancer referrals, nor the length of delays in A&E units had any effect on levels of trust in the NHS. One difference across the two models is gender: women experience lower levels of trust in the NHS in Model 2. The two ordered logistic models presented in the Appendix also both find a negative relationship with women and trust in the NHS. The level of Conservative vote in the constituency also achieves a negative (same direction) relationship with trust in the NHS. The significance/non-significance of all other factors holds constant. Wave fixed effects were also tested and made no difference to any of our findings. This strengthens our confidence in the robustness of our main results.

**Table 5:** Regression model of levels of trust in the NHS.

	Dependent variable	
	Trust in the NHS	
	(1)	(2)
Cancer referral delay	0.239	0.169
	(0.197)	(0.172)
Hospital A&E delay	0.331	−0.012
	(0.347)	(0.305)
Woman	−0.054	−0.076**
	(0.042)	(0.036)
Income	0.0001	
	(0.007)	
Age	0.008***	0.007***
	(0.001)	(0.001)
Member of ethnic minority	−0.395***	−0.483***
	(0.064)	(0.054)
Higher education	−0.024	0.015
	(0.045)	(0.037)
Voted Conservative in 2019	−0.415***	−0.388***
	(0.046)	(0.041)
Constituency unemployment rate	−0.043***	−0.041***
	(0.016)	(0.014)
Conservative vote share 2019	−0.281	−0.210
	(0.191)	(0.168)
North West	0.018	0.022
	(0.106)	(0.093)
Yorkshire and the Humber	0.160	0.155
	(0.109)	(0.097)
East Midlands	0.010	0.045
	(0.114)	(0.101)
West Midlands	−0.031	0.004
	(0.116)	(0.104)
East of England	−0.068	0.014
	(0.117)	(0.103)
London	0.069	0.134
	(0.108)	(0.095)
South East	0.029	0.068
	(0.105)	(0.093)
South West	0.065	0.084
	(0.118)	(0.105)
(Intercept)	4.963***	5.000***
	(0.197)	(0.169)
Observations	5047	6706
R2	0.033	0.033

Note: *p < 0.1; **p < 0.05; ***p < 0.01.

## 4 Discussion

The main finding in this paper is that respondents’ trust towards the NHS is not significantly influenced by localised regional waiting times: neither cancer waiting times nor A&E waiting times had a significant effect on trust. Another finding was that both ethnic minority respondents and Conservative voters had approximately 0.4 points less trust in the NHS on average than white or non-Conservative voters in contrast.

A recent study on the NHS suggests that the British people love the institution [2]. This might explain a willingness to overlook performance issues on healthcare, which has been established to have a negative impact on healthcare trust in other countries [14]. The findings that wait times have no significant impact on the trust towards the institution challenges other findings, which questions the public support for the NHS [28]. To some extent this is to be expected, as the measure used here is about *trust* in the NHS, while other findings focus on support [28]. In other words, it is quite possible for the public to have decreasing support for the NHS, which could be linked to the performance measures, while still maintaining overall trust in the institution of the NHS. The results here nevertheless suggest that the relationship between performance in terms of regional waiting times and public views towards the NHS should be further examined.

In political terms the NHS, and welfare services in general, are often considered as issues on which there is broad agreement to support, yet it is argued that under-funding and significant, and often negatively viewed, reform of the NHS often happens under Conservative governments [29,30]. Finding a stark difference of 0.4 points (of a 7-point scale) in trust towards the NHS between Conservative and non-Conservative voters does suggest a strong political divide between voters. A divide that is somewhat surprising as it is also well-established that Conservative voters in the UK tend to be older than average which makes them more likely to be users of the NHS. Age itself has a small positive effect, while there is no statistically significant effect of income on trust in the NHS. The political differences in trust on the health services are of a nature where it is possible to question whether it is truly a “National” Health Service: when 37% of the respondents has an average half a point lower trust towards such a central institution, being able to measure trust is important for the efficient delivery of healthcare [15,16].

A similar worrying finding is the significantly lower trust in the NHS among ethnic minorities, also of about 0.4 points of a seven-point scale. That there are health inequalities based on ethnicity is not new [31,32]. If anything, this can be seen as a further performance indicator in that it is not disputed that particular ethnic groups have unequal access and experience with the NHS which in turn may explain the stark difference between ethnic minority respondents and white respondents. Ethnic minorities account for 14% of the respondents in the surveys used and with strong variation across the country: London respondents are 40% ethnic minorities versus 60% white, while in the North East of England minorities only account for 4% of the respondents. The problem with having lower levels of trust among a particular group, which in some parts of the country is a very sizeable group, is that the existing health inequalities can be exacerbated if trust levels are lower.

Consistent with cross-national research is our finding that increased unemployment is associated with less trust in the health services. Where employment decreases, so does trust in the NHS. The negative relationship between health and unemployment is long-established [33,34] and in areas where unemployment spikes there tends to be a similar increase in self-reported poor health.

The findings in this paper provide strong evidence that trust in the NHS is determined by sociodemographic and political variables instead of regional waiting times. This can inform policymakers and stakeholders in addressing reforms of and interventions in the NHS.

## 5 Study limitations

Participants were recruited through the YouGov panel and as such those in the population who are not using the internet are not represented in this study. These groups tend to be those who are perhaps not able to do so due to economic factors or age; however, we do not believe that any group is systematically underrepresented in the sample. From the discussion we must also mention that we focused on trust and not support; these are not two sides of the same coin and we cannot rule out that a different result could be reached by having a different question for measuring trust. Given the goal was a nationally representative sample the number of ethnic minority respondents fits this, although the findings we present here do suggest the need for further exploration of the trust in the NHS among ethnic minorities, but that will require a substantially different sample.

We also have to point out that we do not capture whether the respondents have had any *personal* experience with wait times in A&E or Cancer units. This does pose a limitation on our conclusions, although we also note that the extended media coverage of NHS waiting times makes it likely that most people will have an idea about the potential issue. We are not ruling out that there is a possibility that own experience could influence the findings in a particular fashion, although as presented in Fig. 1 the overall trust in the NHS is very high compared to other institutions.

## 6 Conclusion

The paper examined the effect of waiting times on trust in the NHS by using a nationally representative survey of over 6600 respondents with data collected over a year. We find no effect of waiting times of respondents’ trust towards the NHS, but instead find strong negative relationships for ethnic minorities, levels of unemployment and Conservative voters.

The study presents evidence for the NHS, politicians and other stakeholders in the NHS of where to focus efforts to increase trust in a crucial public institution in England. Trust in the health services is crucial for a welfare state and inequality in how the trust is distributed can further exacerbate existing inequalities.

It is necessary to take these differences seriously when considering the role of the NHS in England, despite the love that appears to be present for the NHS [2], though love might not equal trust.

## Ethical approval

The data collected in this study were gathered anonymously through the YouGov panel. The researchers followed all regulations present in England concerning ethics and data protection. Approval was granted by Brunel University London, reference number 35290-LR-Jan/2022-37313-1.

## Funding

This study was funded by a joint award from the 10.13039/100014013UKRI/ESRC (10.13039/501100000269Grant reference ES/W011913/1) and the JSPS (Grant reference JPJSJRP 20211704).

## Data availability

Replication code and data are available through the Harvard Dataverse at: https://doi.org/10.7910/DVN/AQYYNK.

The data are in the public domain, under the terms of the Creative Commons CC0 1.0 Universal deed.

## Declaration of competing interests

The authors declare that they have no known competing financial interests or personal relationships that could have appeared to influence the work reported in this paper.

## Figures and Tables

**Table A1:** Ordered logistic regression model using cumulative link models

	Dependent variable	
	as.factor (trustNHS)	
	(1)	(2)
Cancer referral delay new	0.231	0.219
	(0.246)	(0.211)
Hospital A&E delay	0.450	−0.109
	(0.432)	(0.374)
Woman	−0.108**	−0.140***
	(0.052)	(0.044)
Income	0.003	
	(0.008)	
Age	0.011***	0.010***
	(0.002)	(0.001)
Member of ethnic minority	−0.471***	−0.582***
	(0.080)	(0.067)
Higher education	−0.067	−0.004
	(0.055)	(0.045)
Voted Conservative in 2019	−0.521***	−0.480***
	(0.058)	(0.050)
Constituency unemployment rate	−0.052**	−0.050***
	(0.020)	(0.017)
Conservative vote share 2019	−0.464**	−0.346*
	(0.237)	(0.206)
North West	−0.062	−0.015
	(0.135)	(0.117)
Yorkshire and the Humber	0.062	0.125
	(0.138)	(0.121)
East Midlands	−0.031	0.028
	(0.145)	(0.126)
West Midlands	−0.111	−0.034
	(0.148)	(0.129)
East of England	−0.115	−0.003
	(0.149)	(0.128)
London	−0.009	0.106
	(0.137)	(0.118)
South East	−0.004	0.052
	(0.134)	(0.116)
South West	−0.001	0.052
	(0.150)	(0.131)
Observations	5015	6702
Log Likelihood	−8464.052	−11,393.560

Note: *p < 0.1; **p < 0.05; ***p < 0.01.
